# Sampling for SARS-CoV-2 Aerosols in Hospital Patient Rooms

**DOI:** 10.3390/v13122347

**Published:** 2021-11-23

**Authors:** Morgan A. Lane, Maria Walawender, Andrew S. Webster, Erik A. Brownsword, Jessica M. Ingersoll, Candace Miller, Jesse Waggoner, Timothy M. Uyeki, William G. Lindsley, Colleen S. Kraft

**Affiliations:** 1 Division of Infectious Diseases, Department of Medicine, Emory University, Atlanta, GA 30322, USA; andrew.scott.webster@emory.edu (A.S.W.); erik.a.brownsword@emory.edu (E.A.B.); jesse.waggoner@emoryhealthcare.org (J.W.); colleen.kraft@emory.edu (C.S.K.); 2 Rollins School of Public Health, Emory University, Atlanta, GA 30322, USA; maria.walawender@emory.edu; 3 Department of Infectious Diseases, Atlanta VA Health Care System, Decatur, GA 30033, USA; 4 Department of Pathology and Laboratory Medicine, Emory University, Atlanta, GA 30322, USA; jingers@emory.edu (J.M.I.); candace.miller@emory.edu (C.M.); 5 Emory Healthcare, Atlanta, GA 30322, USA; 6 Influenza Division, National Center for Immunization and Respiratory Diseases, Centers for Disease Control and Prevention, Atlanta, GA 30322, USA; tmu0@cdc.gov; 7 National Institute for Occupational Safety and Health, Centers for Disease Control and Prevention, Morgantown, WV 26508, USA; wdl7@cdc.gov

**Keywords:** COVID-19, airborne disease, viral aerosols, hospital, sampling

## Abstract

Evidence varies as to how far aerosols spread from individuals infected with SARS-CoV-2 in hospital rooms. We investigated the presence of aerosols containing SARS-CoV-2 inside of dedicated COVID-19 patient rooms. Three National Institute for Occupational Safety and Health BC 251 two-stage cyclone samplers were set up in each patient room for a six-hour sampling period. Samplers were place on tripods, which each held two samplers at various heights above the floor. Extracted samples underwent reverse transcription polymerase chain reaction for selected gene regions of the SARS-CoV-2 virus nucleocapsid. Patient medical data were compared between participants in rooms where virus-containing aerosols were detected and those where they were not. Of 576 aerosols samples collected from 19 different rooms across 32 participants, 3% (19) were positive for SARS-CoV-2, the majority from near the head and foot of the bed. Seven of the positive samples were collected inside a single patient room. No significant differences in participant clinical characteristics were found between patients in rooms with positive and negative aerosol samples. SARS-CoV-2 viral aerosols were detected from the patient rooms of nine participants (28%). These findings provide reassurance that personal protective equipment that was recommended for this virus is appropriate given its spread in hospital rooms.

## 1. Introduction

Severe acute respiratory syndrome coronavirus 2 (SARS-CoV-2) appears to spread through respiratory droplets and aerosols exhaled from infected individuals [1,2,3]. Infection prevention and control protocols instituted in healthcare facilities during the Coronavirus disease 2019 (COVID-19) pandemic helped reduce the transmission of SARS-CoV-2 [4,5], the virus that causes COVID-19 [6]. It is important to understand how aerosols containing viral RNA spread within a patient room to ensure that personal protective equipment for healthcare workers is appropriate for the setting.

Studies of aerosols in hospital settings have produced varying results, with some studies finding evidence of aerosols containing SARS-CoV-2 RNA in COVID-19 patient rooms and corridors, [7,8,9,10,11,12,13] and others finding little or no evidence of aerosols in the same locations, possibly due to very high air exchange rates [14,15,16,17,18,19,20,21,22,23]. When studies have examined the presence of replication-competent SARS-CoV-2 present in aerosols, most have found that viability is low [24,25,26]. This may reflect difficulty in isolating SARS-CoV-2 using available viral culture methods or that most individuals with COVID-19 had decreased infectiousness during hospitalization [26,27]. Viral culture utilized to quantify SARS-CoV-2 present in exhalations from infected individuals has found that the viral load in exhaled breath increases in proportion to disease severity [28,29,30]. Other individual factors that influence disease severity, such as obesity and other co-morbidities, may also impact the infectiousness of a patient and their ability to aerosolize viral droplets [28]. The size of the exhaled particles, and the resulting ability to spread at various distances, are also known to impact the ability of the virus-containing aerosols and droplets to cause infection in an individual, with various particles sizes travelling to different areas of the respiratory system [31,32]. It is not yet known what the infectious dose or the most effective particle size is for transmission SARS-CoV-2 transmission, but it seems that smaller particles remain suspended in the air for longer and are better able to travel to the lungs [31,33]. It is also clear that containment measures, such as social distancing and mask mandates, were effective in reducing the transmission of the virus, giving insight into dispersion dynamics of virus-containing aerosols [34].

To determine the spread of aerosols containing SARS-CoV-2 viral RNA inside hospital rooms of patients with COVID-19, we used National Institute for Occupational Safety and Health (NIOSH) bioaerosol samplers placed at various distances from the patient to evaluate the presence of SARS-CoV-2 in aerosols at multiple locations. We collected data on the clinical characteristics of participants to gain a better understanding of whether these characteristics could impact detection of aerosols containing viral nucleic acid.

## 2. Materials and Methods

This study took place on dedicated COVID-19 patient care units at a tertiary care academic medical center from 7 January to 27 May 2021. Patients aged 18 years and older were eligible for room sampling within 24–48 h of hospital admission for COVID-19 confirmed by reverse transcription polymerase chain reaction (RT-PCR) assay for SARS-CoV-2 in respiratory specimens. A convenience sample of participants was used, with newly admitted patients chosen for sampling. SARS-CoV-2 testing at our institution during this timeframe included the Cepheid (Sunnyvale, CA, USA) GeneXpert Xpress SARS-CoV-2 and Xpress SARS-CoV-2/Flu/RSV (positive Cycle threshold (Ct): <45), Roche (Rotkreuz, Switzerland) cobas 6800 SARS-CoV-2 (positive Ct: 36/37), and Diasorin Molecular (Cypress, CA, USA) Simplexa COVID-19 (SIMCOVID) Direct tests (positive Ct: 36/37). Participants provided verbal informed consent as approved by the Emory University Institutional Review Board (STUDY00000466). Patients requiring mechanical ventilation at the time of sampling were excluded from the study.

### 2.1. Patient Data 

Clinical characteristics were collected for each participant from the electronic medical chart for the time of sampling, including days since symptom onset, COVID-19 related treatments administered and duration of therapy, ambulatory status, use of oxygen, whether they were diagnosed with pneumonia (by radiology or clinical definition), whether they were coughing on the day of sampling, and comorbidities such as hypertension, diabetes, and COPD. The Ct value from the NP swab taken on the day of admission was also collected. Participant characteristics were compared between those whose rooms had positive versus negative aerosol samples using a two-sample *t*-test for continuous variables and a chi-square test for categorical variables at an alpha level of 0.05.

### 2.2. Sampling Set-Up

**Figure A1:**
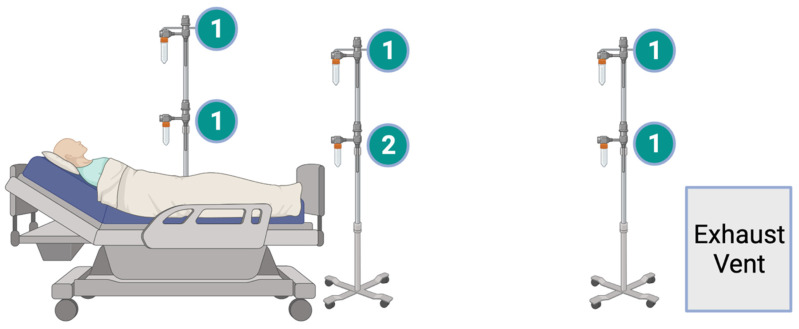
Locations of the aerosol samplers in patient rooms.

Six NIOSH (Morgantown, WV, USA) BC 251 two-stage cyclone samplers were used for sampling [35,36]. These NIOSH samplers separate particles into three size fractions, which are collected in a 15 mL centrifuge tube (>4 µm), a 1.5 mL centrifuge tube (1–4 µm), and on a filter cassette containing a 37-mm diameter polytetrafluoroethylene filter with 2 µm pore size (<1 µm). Samplers were connected with 6.35-mm Tygon tubing to a sampling pump (PCXR-4, SKC, Eighty-Four, PA, USA) at a 3.5 L/min flow rate. Two samplers were placed inside each participant’s room at 1 m and 1.5 m, respectively, above the floor on each of three tripods to approximate the height of a patient’s head in bed and of the exhaust vent. One tripod was placed by the head of the patient bed (~0.3 m from head), one centered at the foot of the bed (~2 m), and one near the exhaust vent for the room (~3 m) (Figure A1), similar to a previous study we conducted inside of a patient room [22]. The specific distance from the participant may have differed slightly in each room as samplers could not interfere with patient care equipment, but samplers were placed in the same general locations. Air exchanges in the sampled rooms were not measured, as none were airborne isolation or negative pressure rooms. We estimate air exchanges occurred 2–6 times per hour, depending on the room, and this rate was likely constant within each room [37]. The samplers were run for a six-hour period.

### 2.3. Aerosol Samples Processing

At the end of the sampling period, all aerosol samples were immediately stored dry at −80 °C in the laboratory where the samples were processed. Due to supply constraints during the pandemic, aerosol samples were tested using two different methods, and some sample processing used sterile phosphate buffered saline (PBS) as an alternative to viral transport media (VTM). The first 172 samples were processed with VTM, with the remaining 404 processed with PBS. This medium change had been validated as an adequate substitution in Emory Medical Laboratories. Collected samples were processed in a biosafety level II cabinet as follows: (1) 1000 µL VTM (Copan UTM, Murieta, CA, USA) or PBS was added to the 15 mL tube, the tube was vortexed, inverted, and vortexed again, then frozen at −80 °C; (2) 400 µL VTM or PBS was added to the 1.5 mL tube, the tube was vortexed, inverted, and vortexed again and frozen at −80 °C; (3) sterile forceps were used to remove the filter from its cassette for placement in a 15 mL tube, 1000 µL of VTM or PBS was added to wet the entire filter, and the tube was vortexed and stored at −80 °C.

### 2.4. Viral RNA Isolation

RNA extraction was performed using the m2000 (Abbott Molecular, Abbott Park, IL, USA) with 600 µL of input sample volume, and sample elute of 50 µL. All extracts were then frozen at −20 °C or colder until amplification.

### 2.5. Reverse Transcription Polymerase Chain Reaction

For samples 1–112, extracted samples were thawed and then were tested by real-time reverse transcription polymerase chain reaction (rRT-PCR) by a triplex laboratory developed test (LDT) targeting N2 and the envelope (E) gene of SARS-CoV-2 and the RNAase P gene (PMC7323516) [38].

Samples 113–576 were tested by rRT-PCR for selected gene regions of the SARS-CoV-2 virus nucleocapsid (N1, N2) and human RNase P gene (IDT 2019-nCoV RUO Kit, Integrated DNA Technologies) using a protocol adapted from the Centers for Disease Control and Prevention (CDC) [39]. Following RNA extraction, 20 µL reactions were set up containing 5 µL of sample RNA and one of two reaction mixes, based on reagent availability. The first reaction mixture was prepared with 8.5 µL of nuclease free water, 1.5 µL of combined primer/probe mix, and 5 µL of TaqPath™ 1-Step RT-qPCR Master Mix (Thermo Fisher, Waltham, MA). Thermal cycling was performed at 25 °C for 2 min followed by 50 °C for 15 min, followed by an initial denaturation at 95 °C for 2 min, followed by 45 cycles of amplification at 95 °C for 3 s, and 55.0 °C for 31 s (Thermo Fisher 7500, Waltham, MA, USA).

A control plasmid containing the complete nucleocapsid gene from 2019 nCoV-2, IDT nCoV-N, was extracted and tested concurrently as a positive control on all runs. Exponential growth curves that crossed the threshold line within 40 cycles [Ct value < 40] were considered positive. The limit of detection for samples 1–112 was 5 viral copies/µL. For samples 113–576, it was 1 viral copy/µL.

## 3. Results

### 3.1. Patient Characteristics

Throughout January–May 2021, 32 participants were enrolled, and their rooms sampled (Table 1). All participants were hospitalized on general medical wards and ranged in age from 26 to 101, with an average age of 55 years. Thirty participants (94%) had co-morbidities with the most common being hypertension (*n* = 15, 45%) and diabetes mellitus (*n* = 12, 38%). Included participants had experienced symptoms for a mean of 8.5 days at the time of sampling, 25 patients (78%) were ambulatory, 15 (47%) required supplemental oxygen, and 24 (78%) were receiving therapeutic agents (e.g., dexamethasone or remdesivir) at the time of sampling. The assays used to test the NP swabs upon admission varied across participants, according to what was available at the time. Twenty-nine samples were tested on the GeneXpert assay, one was tested on the SIMCOVID assay, and two were tested on the Roche cobas 6800 assay. For samples run on an assay other than the GeneXpert, the type of assay is noted in Table 1. Ct values from the NP swabs run on the GeneXpert assay ranged from 18.8 to 44.4 (median Ct value = 25.5, SD = 6.76).

### 3.2. Aerosol Samples

Out of 576 aerosol samples collected, 19 (3%) were positive for SARS-CoV-2 RNA from the rooms of nine participants. Seven of the positive samples were collected in the room of one participant who had an admission NP swab with a Ct value of 21.3 (Participant 20). Two of these positive samples were collected at the head of the bed (one: high sampler; one: low), three at the foot of the bed (one: high; two: low), and two at the exhaust vent (one: high; one: low) (Appendix A). All but one of these positive samples was collected in the 1.5 mL tube, with the last sample collected in the filter. Overall, eight positive samples were collected from the sampler at the head of the bed, nine from the sampler at the foot of the bed, and two from the sampler near the exhaust vent (Table 2). Most positive samples were collected in the 1.5 mL tube, most commonly at the foot of the bed (Table 2). Only one positive sample was collected by the filter, and two by the exhaust vent.

### 3.3. Statistical Analysis

Three participants (17, 25, and 28) were excluded from statistical analysis because they originally tested positive for SARS-CoV-2 at least one month prior to sampling and were hospitalized at the time of sampling for non-COVID diagnoses. Participant 17 was experiencing pneumonia upon hospitalization and tested positive four months prior to sampling. This participant was assumed not to be experiencing pneumonia due to COVID-19; however, a positive aerosol sample was detected in their room at the foot of the bed, indicating the possibility of reinfection or persistent infection, though the Ct value from the nasal swab was very high (44.4), indicating low viral load. Participants 25 and 28 were not experiencing COVID-19 symptoms at the time of sampling and were enrolled because they had a NP swab that tested positive for SARS-CoV-2 on admission, which may have reflected persistent viral RNA detection and not acute infection but were later excluded after chart review revealed the earlier positive test.

For the remaining participants whose samples were run on the GeneXpert assay, those with positive aerosol samples did not have significantly different Ct values (M 26.8, SD 7.9) than those with negative aerosol samples (M 27.6, SD 1.5), *p* = 0.17. Days since symptom onset were slightly lower for participants with positive samples (M 6.9, SD 2.1) versus negative (M 9.3, SD 5.3) though not significantly lower, *p* = 0.09.

At the time of sampling, most participants were receiving treatment with dexamethasone and remdesivir (*n* = 18, 56%), one was receiving dexamethasone alone, one was receiving remdesivir alone, four were receiving corticosteroid tapers following treatment with dexamethasone and remdesivir, and two had received anti-SARS-CoV-2 monoclonal antibodies. All but one positive aerosol sample were from participants receiving multiple therapies, and one from a participant who had received monoclonal antibodies. For participants who received any treatment (dexamethasone, remdesivir, or both), duration of treatment for participants with negative aerosol samples (M 3.0, SD 3.0) was slightly longer than those with positive samples (M 1.6, SD 0.5), though not statistically significantly different *p* = 0.14.

No differences were observed between participants with positive versus negative aerosol samples whether they were ambulatory (*p* = 0.31), on supplemental oxygen (*p* = 0.34), receiving therapeutics (*p* = 0.24), diagnosed with pneumonia (*p* = 0.64), coughing (*p* = 0.73), or whether they had co-morbidities (*p* = 0.09). There was no difference between patients with SARS-CoV-2 positive versus negative aerosol samples whether they had diabetes (*p* = 0.50), hypertension (*p* = 0.29), or obesity (*p* = 0.94), the most common co-morbidities.

## 4. Discussion

Of 576 aerosol samples collected from 19 different rooms across 32 participants, only 3% (9) were positive for SARS-CoV-2 by rRT-PCR. The small number of positive samples limited our ability to identify risk factors for the presence of SARS-CoV-2 in aerosol samples. The Ct values of the NP swabs and participant comorbidities did not vary significantly between participant in rooms where SARS-CoV-2 positive aerosols were detected compared with those in rooms where no SARS-CoV-2 aerosols were detected. This was somewhat unexpected, as previous studies have shown that the amount of exhaled SARS-CoV-2 particles varies based upon disease severity and the co-morbidities of the infected individual [1,28]. In this study, however, all but three participants had comorbidities, making comparison across the groups difficult. The duration of participants symptoms and treatments did appear to be shorter for participants with positive aerosol samples, though not at a statistically significant level.

It is possible that by the time most of the participants in this study were hospitalized, the concentration of exhaled SARS-CoV-2 particles were undetectable with our aerosol sampling methods. The average time from symptom onset to aerosol sampling in our participant population was 8.5 days, which may be beyond the point at which viral nucleic acid begins to decrease. However, the median Ct values for the participants was <30, which may indicate possible infectiousness at the time of sampling [40,41]. The participant in the room where multiple positive aerosol samples were collected had been symptomatic for 8 days with an admission NP swab that yielded a Ct value of 21.3, suggesting high viral load. In two similar studies that conducted aerosol sampling in patient rooms, positive aerosol samples were only identified in the rooms of patients within 2–5 days of symptom onset [7,12]. Another study, however, found that positive samples were more frequently identified later in the course of illness. This study used a high-volume sampler with an air flow rate of 50 L/min that may have improved detection [10]. In our study, we ran low-volume samplers (3.5 L/min) for six hours to account for the lower air flow rate.

Limitations of this study include the small number of SARS-CoV-2 positive aerosol samples, inability to assess the infectiousness of SARS-CoV-2 in positive samples, use of different processing procedures and assays, and potential differences in room air flow and size. The percentage of positive aerosol samples was 3%, which limited our statistical analyses, especially because a single participant contributed 37% of the positive aerosol samples. Further, the participants included in this study only represent a portion of the possible clinical representations of COVID-19 given none were intubated, all were on general medical wards, and some were ambulatory. Thus, these results may not be generalizable to other patient populations or healthcare settings. Since we did not perform viral culture, it is unknown whether any positive aerosols contained infectious virus and represented a transmission risk. Additionally, it is possible that the reason for the small number of SARS-CoV-2 RNA-positive samples detected in this study is that they may have been below the limit of detection of 10 viral copies/mL. It is unclear if such low viral RNA levels would pose a transmission risk.

Aerosol concentrations within rooms can vary greatly and are impacted by the air flow in the room. The rooms in which we sampled differed in size and likely differed in the number of air exchanges per hour, which may have influenced the ability to detect aerosols. However, we detected positive aerosol samples in a variety of rooms. Aerosol samplers also only collect aerosols from the immediate vicinity of the sampler and may have missed larger aerosols present in other locations in the room that were too far from a sampler. However, we intentionally placed three samplers in the room at increasing distances from the participants to minimize the risk of missing the presence of aerosols. It is also possible that viral RNA could have degraded within the sampler; however, a recent study suggests that this is unlikely [42].

The results of this study further our understanding of the dispersion dynamics of SARS-CoV-2 in hospital rooms, with implications for healthcare provider and patient safety. Although our findings suggest that SARS-CoV-2 detection in aerosols within the rooms was minimal, we did detect viral RNA in some patient’s rooms. We do not know the infectiousness of the aerosols in which a low frequency of SARS-CoV-2 RNA was detected; however, given there is demonstrated spread of aerosols containing viral RNA, our findings are reassuring that the current CDC respiratory protection recommendations for healthcare personnel to wear fit-tested N95 or equivalent or higher level respirators and eye protection in addition to gown and gloves when caring for patients with suspected or confirmed SARS-CoV-2 infection are appropriate [43].

## Figures and Tables

**Table 1:** Summary of participant characteristics at the time of sampling.

Group	No. per Group	Avg. Days since Symptom Onset	Ambulatory	Rec. Oxygen	Coughing	Co-Morbid. Present ^1^	Pneumonia	Rec. Treat. (D & R) ^2^	Avg. Treat. Duration ^3^	Avg. Ct Value of NP Swab ^4^
Total	32	8.5	25	15	14	28	21	24	4.4	27.3
Positive Aerosol Samples	9	6.6	6	6	5	9	6	7	1.6	26.8
Negative Aerosol Samples	23	9.3	19	9	9	19	15	15	3.6	27.6

^1^ Co-morbidities include diabetes, heart transplant, COPD, hypertension, and others; ^2^ Therapies included: D: dexamethasone; R: remdesivir, D and R together; ^3^ Only including those treated with D, R, or D + R; ^4^ Ct values for those tested on the GeneXpert only (20 in the negative group and 9 in the positive group).

**Table 2:** Number of positive aerosol samples from each sampler location and collection vessel.

Location	High Sampler		Low Sampler		
	15 mL	1.5 mL	15 mL	1.5 mL	Filter
Head of bed	1	4	1	2	0
Foot of bed	0	6	0	2	1
Exhaust Vent	0	1	0	1	0

## Data Availability

The data presented in this study are available on request from the corresponding author. The data are not publicly available due to restrictions with sharing patient information.
